# Association of advanced lung cancer inflammation index with hypertension in obstructive sleep apnea

**DOI:** 10.1097/MD.0000000000050538

**Published:** 2026-09-11

**Authors:** Mengying Lin, Yuxin Yang, Zongshan Li, Tian Lv, Yourang Pan, Songtao Li, Shaoting Yu, Xiaoyan Yang, Jia Huang

**Affiliations:** a Department of Psychosomatic, Shaoxing Seventh People’s Hospital, Affiliated Mental Health Center, Medical College of Shaoxing University, Shaoxing, Zhejiang, China; b College of Medical, Shaoxing University, Shaoxing, Zhejiang, China; c Department of Neurology, Xuanwu Hospital, Capital Medical University, Beijing, China; d Department of Neurology, Zhuji Affiliated Hospital of Wenzhou Medical University, Zhuji, China; e Department of Psychiatry, Shaoxing Seventh People’s Hospital, Affiliated Mental Health Center, Medical College of Shaoxing University, Shaoxing, Zhejiang, China; f Department of Otorhinolaryngology, Zhuji Affiliated Hospital of Wenzhou Medical University, Zhuji, China.

**Keywords:** Advanced Lung Cancer Inflammation Index (ALI), Hypertension, NHANES, obstructive sleep apnea (OSA)

## Abstract

The relationship between obstructive sleep apnea (OSA) and hypertension has been well established. The Advanced Lung Cancer Inflammation Index (ALI), which integrates markers of inflammation and nutritional status, has been associated with hypertension in OSA patients. This study aimed to explore the association between ALI and hypertension among individuals with OSA. Data from 12,049 participants across four National Health and Nutrition Examination Survey (NHANES) cycles were analyzed. OSA was identified using a questionnaire-based sleep-related information. Logistic regression models adjusted for confounders and restricted cubic spline (RCS) analyses were conducted to evaluate the associations and potential nonlinear relationships. Logistic regression analysis showed that each unit increase in Log ALI was associated with higher odds of hypertension (OR = 1.44, 95% CI: 1.25–1.66, *P* < .001). Quartile analysis demonstrated that participants in the fourth ALI quartile had higher odds of hypertension than those in the lowest quartile (OR = 1.70, 95% CI: 1.40–2.07, *P* < .001). RCS analysis supported the nonlinear association between ALI and hypertension. This study suggests a significant association between ALI and hypertension in individuals with OSA, highlighting the potential relevance of inflammation and nutritional status in hypertension.

## 1. Introduction

Obstructive sleep apnea (OSA) is a prevalent chronic disorder characterized by recurrent partial or complete obstruction of the upper airway during sleep, resulting in episodes of apnea and hypopnea.^[1]^ These events cause intermittent hypoxemia, fluctuations in autonomic nervous system activity, and sleep fragmentation.^[2]^ Clinically, OSA presents with symptoms such as loud snoring, disrupted sleep, and excessive daytime sleepiness, which adversely affect the quality of life, cognitive function, and social interactions.^[3]^ The pathophysiology of OSA is multifaceted and involves anatomical abnormalities, functional deficits, obesity, physiological changes, and inflammatory pathways.^[4]^ Globally, OSA affects nearly one billion adults, with a substantial proportion experiencing moderate-to-severe disease associated with significant cardiovascular and metabolic consequences.^[5]^

OSA is recognized as an independent risk factor for several chronic diseases, including diabetes, stroke, and hypertension, and is associated with adverse outcomes.^[6–9]^ Among these, hypertension, linked to OSA, significantly contributes to increased cardiovascular morbidity and mortality. Despite extensive research, the precise mechanisms linking OSA and hypertension remain unclear. In particular, limited evidence is available regarding the role of integrated inflammatory–nutritional markers, such as ALI, in hypertension among individuals with OSA. This knowledge gap highlights the need for further investigation, especially because hypertension in patients with OSA is often difficult to manage and is associated with substantial cardiovascular risk.

Current research highlights a bidirectional relationship between OSA and hypertension, with OSA affecting over 30% of hypertensive individuals and nearly 80% of those with resistant hypertension.^[10,11]^ Approximately half of the patients with OSA develop hypertension.^[12]^ Mechanistically, recurrent apnea and hypopnea episodes trigger sympathetic overactivation, RAAS dysfunction, and systemic inflammation, leading to persistent hypertension.^[13]^ Contributing factors, such as obesity, hypoxemia, and hypercapnia further exacerbate inflammatory responses and elevate blood pressure.^[14,15]^ Treatment-resistant or nocturnal hypertension in OSA is frequently accompanied by vascular remodeling.^[16]^ In addition, unhealthy dietary patterns, particularly high-fat diets, may contribute to gut microbiota dysbiosis and systemic inflammation, thereby exacerbating metabolic abnormalities and OSA severity.^[17]^ These findings further support the importance of investigating inflammatory and nutritional indicators such as ALI in OSA-related hypertension.

The advanced lung cancer inflammation index (ALI) is a composite measure derived from body mass index (BMI), serum albumin levels, and the neutrophil-to-lymphocyte ratio (NLR).^[18]^ Among these components, NLR is recognized as a rapid and reliable indicator of systemic inflammation and immune dysfunction,^[19]^ whereas BMI and serum albumin levels reflect nutritional and metabolic status. By integrating these parameters, ALI may provide a more comprehensive assessment of inflammatory and nutritional status than any single marker alone. Initially developed as a prognostic marker for various cancers,^[20–22]^ ALI has increasingly been recognized as a useful indicator of chronic conditions influenced by inflammation and nutrition.^[23–25]^ Previous studies have suggested that inflammatory and nutritional markers may play an important role in cardiovascular and OSA-related outcomes. Tu et al reported that higher ALI levels were significantly associated with lower cardiovascular mortality in patients with hypertension.^[26]^ In addition, Kadier et al demonstrated significant correlations between OSA symptoms and inflammatory indices, including the systemic immune-inflammation index (SII), NLR, and platelet-to-lymphocyte ratio (PLR).^[27]^ These findings provide a rationale for further investigation of the association between ALI and hypertension in individuals with OSA.

To date, few studies have explored the association between ALI and hypertension among individuals with OSA. Therefore, this study used NHANES data to investigate the relationship between ALI and hypertension in patients with OSA and further evaluate the potential role of inflammation and nutritional status in this population.

## 2. Methods

This study used nationally representative data from the National Health and Nutrition Examination Survey (NHANES) conducted by the National Center for Health Statistics (NCHS) under the Centers for Disease Control and Prevention (CDC). The NHANES dataset included five primary components: demographic information, dietary intake data, physical examination results, laboratory measurements, and survey responses. All study procedures were approved by the NCHS Research Ethics Review Board, and informed consent was obtained from all the participants. Comprehensive details regarding the methodologies and data collection protocols can be accessed on the NHANES website (http://www.cdc.gov/nchs/nhanes.htm).

### 2.1. Population

**Figure 1.**
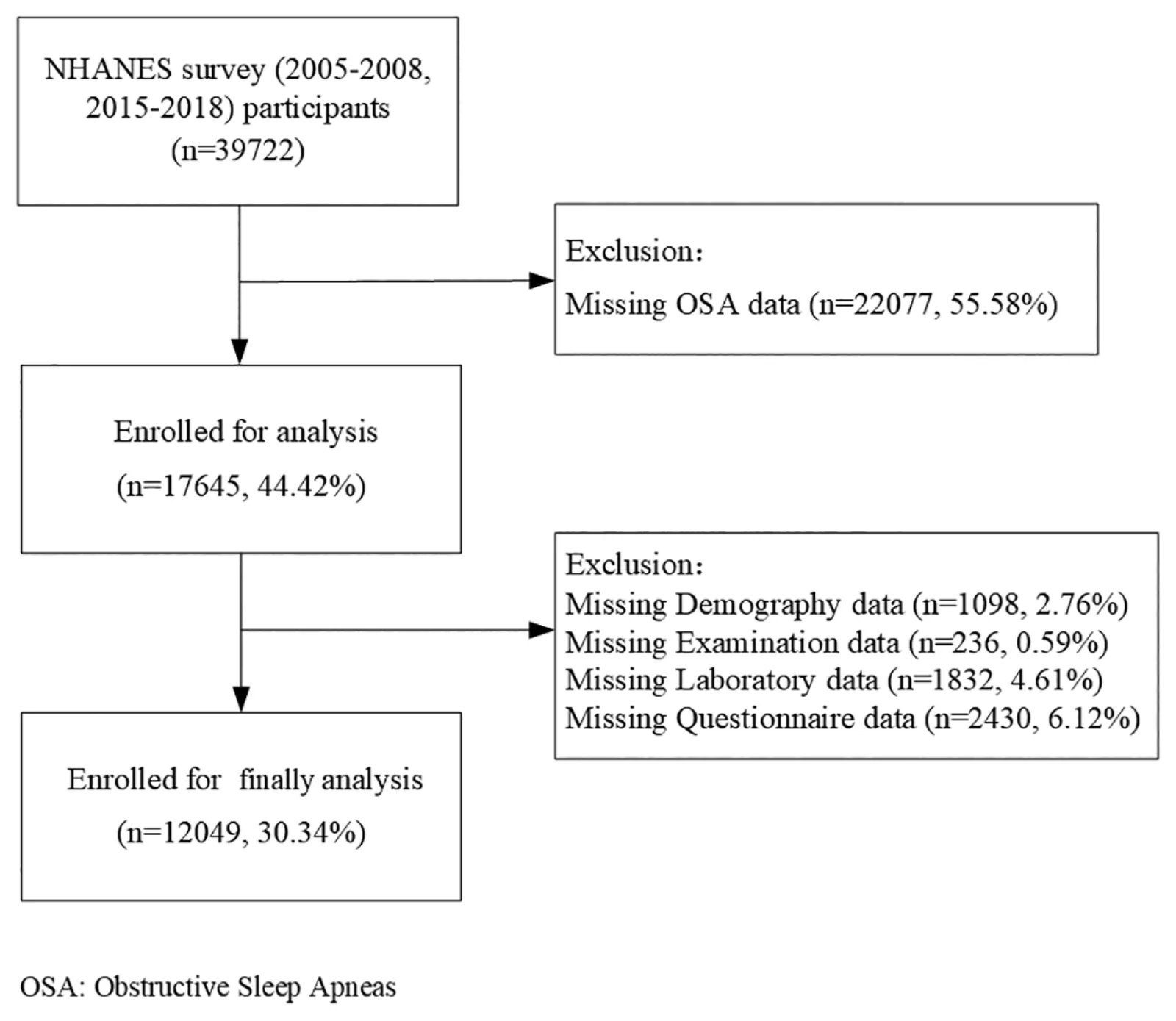
Flow chart of the study population.

This analysis initially included data from 39722 participants across four NHANES cycles (2005–2006, 2007–2008, 2015–2016, and 2017–2018). Exclusion criteria were missing information on key variables, including obstructive sleep apnea (OSA) (n = 22,077), poverty income ratio (PIR) (n = 1097), education (n = 1), body mass index (BMI) (n = 236), serum albumin (n = 220), neutrophil-to-lymphocyte ratio (n = 1610), serum creatinine (n = 2), diabetes (n = 278), smoking status (n = 1188), and alcohol consumption (n = 964). After these exclusions, the final sample comprised 12,049 participants. The exclusion of participants with missing data may have affected the representativeness of the study population and introduced a potential selection bias. (Fig. 1)

### 2.2. Variable assessment

The Advanced Lung Cancer Inflammation Index (ALI) was calculated using the following formula: ALI = BMI × albumin (Alb)/ NLR, where BMI (kg/m^2^) represents body mass index, Alb indicates serum albumin levels (g/dL), and NLR is the neutrophil-to-lymphocyte ratio derived from blood test results.^[28]^

To evaluate obstructive sleep apnea (OSA) symptoms, participants completed a sleep questionnaire consisting of three binary questions: Do you snore three or more nights per week? Have you experienced gasping or apnea episodes on three or more nights per week? Despite sleeping for at least 7 hours on weekdays or work nights, do you feel excessively sleepy during the day 16–30 times per month? Respondents who answered “yes” to any of these questions were classified as having OSA symptoms.^[29]^

Although this symptom-based definition has been widely used in epidemiological studies, it does not replace objective diagnostic methods, such as polysomnography, and may not fully capture the presence or severity of OSA.^[29]^

Hypertension was identified based on self-reported diagnosis, the regular use of blood pressure medications, or clinical measurements indicating an average systolic blood pressure (SBP) of at least 140 mm Hg or an average diastolic blood pressure (DBP) of at least 90 mm Hg.^[30]^

### 2.3. Covariate information

The study incorporated multiple covariates, including age, sex, race, education level, poverty income ratio (PIR), alcohol intake, smoking habits, waist circumference, BMI, diabetes status, and hyperlipidemia status. The poverty income ratio (PIR) was calculated as the ratio of total family income to the official poverty threshold specific to family size, year, and state, as defined by the U.S. Census Bureau.

Diabetes was defined based on self-reported diagnosis, use of insulin or antidiabetic medications, or by satisfying one of the following criteria: fasting blood glucose ≥ 7.0 mmol/L, random blood glucose ≥ 11.1 mmol/L, postprandial glucose ≥ 11.1 mmol/L (oral glucose tolerance test), or glycated hemoglobin level ≥ 6.5%. Hyperlipidemia was determined by self-reported diagnosis, current use of lipid-lowering drugs, or meeting at least one of the following conditions: total cholesterol ≥ 200 mg/dL, triglycerides ≥ 150 mg/dL, low-density lipoprotein cholesterol ≥ 130 mg/dL, or high-density lipoprotein cholesterol < 40 mg/dL for males and < 50 mg/dL for females. Laboratory data, including serum albumin, white blood cell count, lymphocyte percentage, eosinophil percentage, monocyte percentage, and neutrophil-to-lymphocyte ratio (NLR), were also evaluated.

### 2.4. Statistical analysis

Statistical analyses were performed using the R Studio version 4.2.0. To account for the complex multistage probability sampling design of the NHANES, appropriate sampling weights, stratification, and clustering were incorporated into all analyses in accordance with NHANES analytic guidelines. Statistical significance was set at a two-tailed *P*-value < .05. All procedures were performed according to NHANES data analysis guidelines. Continuous variables are reported as means with standard errors (SE), while categorical variables are presented as weighted percentages (%). For continuous data, t-tests were applied to normally distributed variables, and nonparametric tests were used for those that did not meet normality assumptions. Categorical variables were analyzed using the chi-squared test.

ALI was analyzed both as a continuous variable and as a categorical variable divided into quartiles. For continuous analyses, ALI values were natural log-transformed to reduce skewness and improve distributional normality.^[21]^ Multivariable logistic regression models were used to assess the association between ALI and hypertension risk in individuals with OSA, with the results presented as odds ratios (ORs) and 95% confidence intervals (CIs). Three models were developed to control for confounding factors: model 1 adjusted for age and sex; model 2 expanded on model 1 by incorporating education level and race; and model 3 included additional adjustments for smoking, alcohol use, diabetes, hyperlipidemia, white blood cell count, and estimated glomerular filtration rate (eGFR). Restricted cubic spline (RCS) analysis was applied to evaluate the nonlinear relationship between ALI and hypertension risk in the OSA population. Stratified analyses by age and sex were conducted to identify potential interactions, whereas subgroup analyses explored variations in the association between ALI and OSA-related hypertension. Sensitivity analyses were performed to evaluate the robustness of primary findings. Specifically, participants from the 2015 to 2016 and 2017–2018 NHANES cycles were excluded, and the analyses were repeated using only the data from the 2005 to 2006 and 2007–2008 cycles. Logistic regression models were reconstructed using the same covariate adjustment strategy as in the primary analyses.

## 3. Results

### 3.1. Baseline characteristics

Participants were categorized into quartiles based on their ALI values. The mean age of the study population was 47.1 years, and 50.69% were male. Higher ALI quartiles were associated with elevated serum albumin levels and monocyte counts along with reduced white blood cell counts and neutrophilic percentages. Furthermore, participants in the higher quartiles exhibited lower NLR values, as well as higher BMI measurements. The participants with hypertension generally exhibited higher ALI levels and a greater prevalence of adverse metabolic and inflammatory characteristics than those without hypertension. (all *P* < .05). No significant differences were observed in education, PIR, or alcohol intake across quartiles. (Table 1)

**Table 1:** Descriptive baseline characteristics of participants.

Variable	Total (n = 4569)	ALI				*P* value
		Q1	Q2	Q3	Q4	
Age	47.097 (0.370)	50.707 (0.572)	47.028 (0.476)	45.299 (0.422)	45.109 (0.488)	<.0001
Sex						.013
Female	5872 (49.315)	1420 (50.533)	1486 (51.629)	1424 (46.097)	1542 (48.961)	
Male	6177 (50.685)	1595 (49.467)	1525 (48.371)	1588 (53.903)	1469 (51.039)	
Race						<.0001
Black	2456 (10.024)	374 (5.534)	445 (7.161)	551 (8.624)	1086 (20.089)	
Other	4228 (19.961)	930 (15.864)	1123 (20.264)	1187 (22.396)	988 (21.503)	
White	5365 (70.016)	1711 (78.602)	1443 (72.575)	1274 (68.979)	937 (58.407)	
Education						.422
<High school	2787 (14.335)	729 (15.202)	725 (14.798)	660 (12.964)	673 (14.390)	
College	6286 (60.415)	1521 (58.991)	1550 (60.017)	1604 (61.516)	1611 (61.238)	
noncollege	2976 (25.250)	765 (25.807)	736 (25.185)	748 (25.520)	727 (24.373)	
PIR	3.087 (0.045)	3.046 (0.049)	3.126 (0.058)	3.149 (0.068)	3.016 (0.051)	.057
BMI (kg/m2)	29.768 (0.123)	26.505 (0.145)	28.897 (0.174)	30.810 (0.169)	33.317 (0.226)	<.0001
Albumin (g/dL)	4.238 (0.009)	4.204 (0.013)	4.235 (0.009)	4.259 (0.010)	4.255 (0.013)	<.001
Hemoglobin(gr/dL)	14.365 (0.037)	14.265 (0.052)	14.354 (0.049)	14.498 (0.040)	14.337 (0.053)	<.0001
WBC (1000cells/ul)	7.413 (0.043)	7.937 (0.062)	7.524 (0.061)	7.147 (0.052)	6.990 (0.104)	<.0001
Neutrophilic (%)	58.065 (0.149)	67.438 (0.135)	60.276 (0.138)	55.525 (0.127)	47.688 (0.161)	<.0001
Monocyte (%)	8.058 (0.036)	7.711 (0.059)	7.920 (0.060)	8.188 (0.053)	8.464 (0.065)	<.0001
NLR	2.163 (0.017)	3.349 (0.030)	2.194 (0.013)	1.748 (0.010)	1.247 (0.008)	<.0001
Hypertension						.015
No	6668 (60.424)	1629 (59.434)	1727 (62.583)	1744 (62.322)	1568 (56.883)	
Yes	5381 (39.576)	1386 (40.566)	1284 (37.417)	1268 (37.678)	1443 (43.117)	
Hyperlipidemia						.008
No	3375 (29.241)	946 (32.609)	829 (28.169)	801 (28.460)	799 (27.513)	
Yes	8674 (70.759)	2069 (67.391)	2182 (71.831)	2211 (71.540)	2212 (72.487)	
Diabetes Mellitus						<.001
Diabetes Mellitus	2461 (15.379)	633 (16.108)	555 (12.802)	605 (15.018)	668 (17.930)	
IFG	690 (6.113)	182 (6.269)	156 (5.025)	173 (6.386)	179 (6.875)	
IGT	432 (3.183)	122 (3.569)	83 (2.436)	122 (3.641)	105 (3.074)	
No	8466 (75.324)	2078 (74.055)	2217 (79.737)	2112 (74.955)	2059 (72.122)	
Alcohol status						.053
Former	2368 (16.437)	669 (18.160)	534 (15.265)	557 (15.341)	608 (17.076)	
Heavy	2394 (21.350)	571 (21.123)	619 (21.402)	607 (21.269)	597 (21.643)	
Mild	3969 (35.948)	1003 (35.851)	1035 (37.510)	985 (35.096)	946 (35.244)	
Moderate	1837 (17.127)	408 (15.199)	471 (17.584)	499 (19.513)	459 (16.062)	
Never	1481 (9.137)	364 (9.667)	352 (8.240)	364 (8.780)	401 (9.976)	
Smoke status						<.0001
Former	3086 (25.844)	848 (25.871)	787 (25.195)	729 (26.356)	722 (25.970)	
Never	6385 (52.557)	1443 (49.606)	1533 (50.849)	1667 (53.333)	1742 (57.018)	
Now	2578 (21.599)	724 (24.523)	691 (23.956)	616 (20.311)	547 (17.013)	<.0001

ALI = advanced lung cancer inflammation index, BMI = body mass index, IFG = impaired fasting glucose, IGT = impaired glucose tolerance, NLR = neutrophil–lymphocyte ratio, PIR = family income-to-poverty ratio, WBC = white blood cell.

### 3.2. Association between ALI and hypertension in OSA

When analyzed as a continuous variable, in Model 1, which was adjusted for age and sex, the OR for OSA-related hypertension was 1.43 (95% CI: 1.27–1.62, *P* < .001). In Model 2, with further adjustments for education level and race, the OR decreased slightly to 1.34 (95% CI: 1.18–1.52, *P* < .001). In Model 3, which additionally accounted for alcohol consumption, smoking status, white blood cell counts, diabetes, hyperlipidemia, and eGFR, each one-unit increase in ALI was associated with a higher odd of OSA related hypertension (OR = 1.44, 95% CI: 1.25–1.66, *P* < .001).

When ALI was treated as a categorical variable divided into quartiles in Model 1, a progressive increase in hypertension risk was observed across quartiles. Compared to the lowest quartile (Q1, ref), the odds ratios were 1.26 (95% CI: 1.07–1.48, *P* = .01) for Q3 and 1.70 (95% CI: 1.43–2.02, *P* < .0001) for Q4. Model 2 showed similar trends, with ORs of 1.25 (95% CI: 1.06–1.46, *P* = .01) for Q3 and 1.56 (95% CI: 1.31–1.87, *P* < .001) for Q4. In Model 3, the association was stronger, with ORs of 1.20 (95% CI: 1.03–1.39, *P* = .02) for Q2, 1.36 (95% CI: 1.14–1.62, *P* < .001) for Q3, and 1.70 (95% CI: 1.40–2.07, *P* < .001) for Q4, all compared to Q1. These findings highlight a strong, dose-dependent relationship between higher ALI levels and an increased risk of OSA-related hypertension (Table 2).

**Table 2:** Multivariate logistic regression analyses for OSA with hypertension and ALI.

Exposure	Model 1		Model 2		Model 3	
	95%CI	*P*	95%CI	*P*	95%CI	*P*
LogALI	1.43 (1.27,1.62)	<.0001	1.34 (1.18,1.52)	<.0001	1.44 (1.25,1.66)	<.0001
ALI for quartiles						
Q1	ref		ref		ref	
Q2	1.11 (0.95,1.30)	.18	1.11 (0.95,1.29)	.19	1.20 (1.03,1.39)	.02
Q3	1.26 (1.07,1.48)	.01	1.25 (1.06,1.46)	.01	1.36 (1.14,1.62)	<.001
Q4	1.70 (1.43,2.02)	<.0001	1.56 (1.31,1.87)	<.0001	1.70 (1.40,2.07)	<.0001
p for trend		<.0001		<.0001		<.0001

Model 1: adjusted for age and sex.

Model 2: adjusted for crude model, age, sex, education, and race.

Model 3: adjusted for all model 2 variables in addition to smoking status, alcohol status, diabetes, hyperlipidemia, WBC, and eGFR.

**Figure 2.**
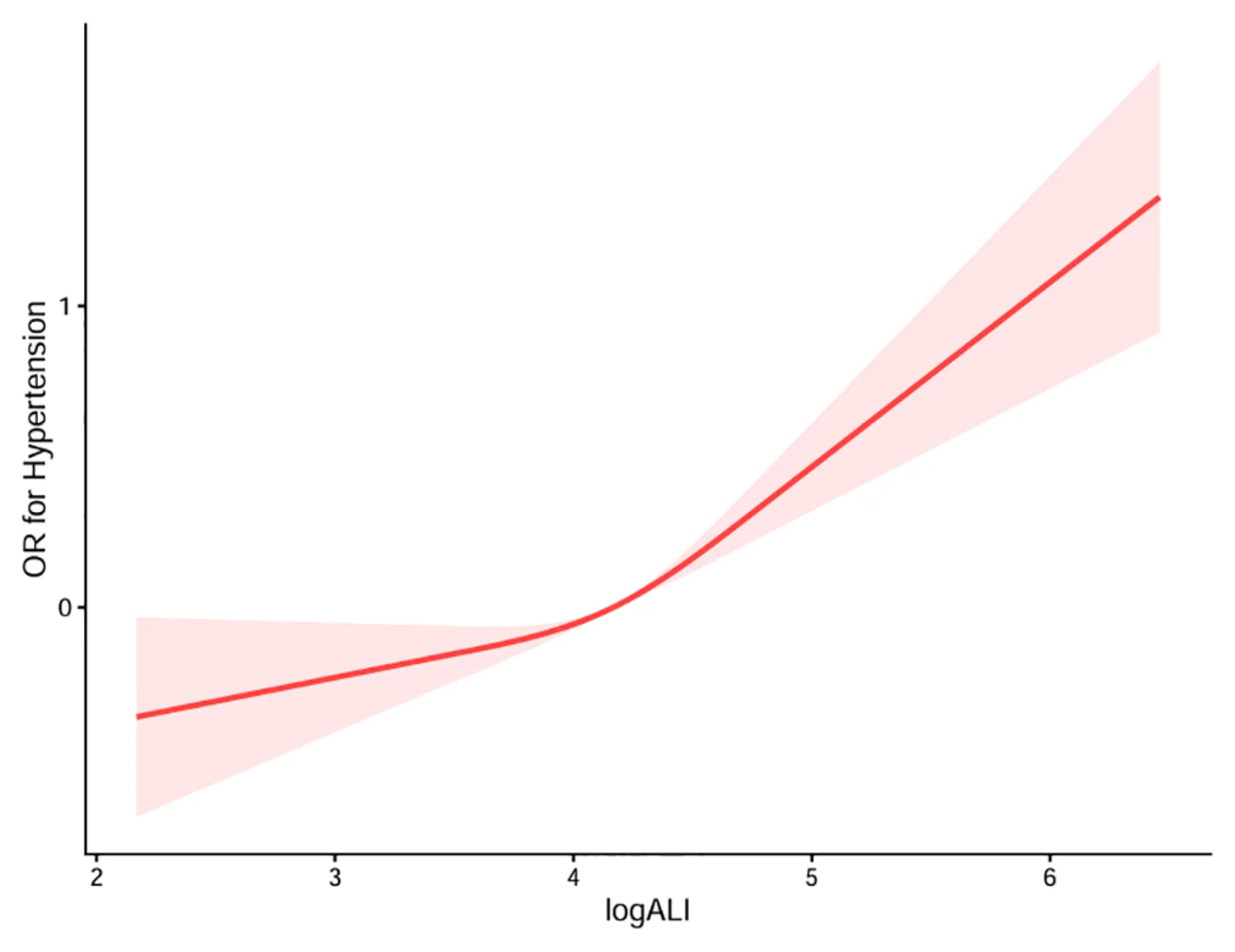
The restricted cubic spline (RCS) curve between ALI and OSA with hypertension.

Restricted cubic spline analysis demonstrated a significant nonlinear association between log-transformed ALI and hypertension (*P* < .001, *P* for nonlinearity = 0.0081). As shown in Figure 2, the odds of hypertension increased progressively with higher ALI levels, and the increase became more pronounced at higher ALI values. This pattern suggests that inflammatory and nutritional status, as reflected by ALI, may have a complex relationship with hypertension in individuals with OSA.

### 3.3. Subgroup analysis

Subgroup analyses indicated no statistically significant differences in the relationship between ALI and hypertension risk across various categories, including age groups (<60 years and ≥ 60 years), education level, smoking habits, alcohol intake, or comorbid conditions, such as diabetes and hyperlipidemia (all *P* > .05). However, sex-specific analysis revealed a significant association between ALI and hypertension risk in female patients with OSA, with an OR of 1.505 (95% CI: 1.071–2.115, *P* = .02). In contrast, no significant association was observed in male patients (OR = 0.829, 95% CI: 0.614–1.120, *P* = .218) (Table 3).

**Table 3:** Subgroup analyses assessing the effect of ALI on OSA with hypertension.

Character	OR (95% CI)	*P*	*P* for interaction	Adjusted *P*
Age			.166	1
>=60	1.14 (0.95, 1.38)	.157		
<60	1.35 (1.16, 1.57)	<.001		
Sex			<.0001	.0007
Male	0.81 (0.68, 0.96)	.017		
Female	1.31 (1.12, 1.54)	.001		
Education			.124	.868
College	1.12 (0.95, 1.32)	.185		
High School	0.93 (0.77, 1.14)	.479		
< High school	0.91 (0.76, 1.09)	.284		
Diabetes Mellitus			.262	1
Diabetes Mellitus	0.87 (0.69, 1.08)	.205		
IFG	1.05 (0.93, 1.19)	.403		
IGT	1.07 (0.79, 1.46)	.654		
No	0.77 (0.44, 1.34)	.341		
Hyperlipidemia			.428	1
Yes	0.96 (0.84, 1.10)	.554		
No	1.06 (0.84, 1.36)	.614		
Smoke status			.236	1
Former	1.11 (0.96, 1.29)	.146		
Never	0.97 (0.77, 1.22)	.798		
Now	0.92 (0.75, 1.12)	.387		
Alcohol status			.271	1
Former	0.98 (0.81, 1.18)	.793		
Heavy	0.92 (0.76, 1.11)	.374		
Mild	1.23 (0.95, 1.60)	.121		
Moderate	1.03 (0.80, 1.32)	.844		
Never	1.17 (0.89, 1.52)	.253		

IFG = impaired fasting glucose, IGT = impaired glucose tolerance.

### 3.4. Sensitivity analyses

Sensitivity analyses yielded results that were generally consistent with the primary analyses. When log-transformed ALI was analyzed as a continuous variable, higher ALI levels were significantly associated with greater odds of hypertension in all adjusted models, including the fully adjusted model (OR = 1.32, 95% CI: 1.10–1.60, *P* = .01).

When analyzed by quartile, participants in the highest ALI quartile exhibited significantly greater odds of hypertension than those in the lowest quartile (Model 3: OR = 1.63, 95% CI: 1.26–2.10, *P* = .002). A significant dose–response relationship was also observed across increasing quartiles (*P* for trend = 0.001). Overall, these findings support the robustness of the association between ALI and hypertension (Table S1, Supplemental Digital Content 1).

## 4. Discussion

This study examined the association between ALI and hypertension among individuals with OSA using the NHANES data. Higher ALI levels were associated with a higher prevalence of hypertension, and this association remained significant after adjusting for potential confounders. Due to the cross-sectional design, causal or predictive inferences cannot be established. Nevertheless, ALI may serve as a clinically accessible inflammatory indicator of hypertension in patients with OSA.

The significant nonlinear association identified by the RCS analysis suggests that the relationship between ALI and hypertension may vary across different ALI levels rather than following a strictly linear pattern. This finding further supports the potential value of ALI as an integrated marker reflecting both inflammatory and nutritional status in individuals with OSA.

The subgroup analyses suggested potential differences in the association between ALI and hypertension across sex subgroups. However, these findings should be interpreted cautiously because subgroup analyses are exploratory in nature and may have been influenced by sample size imbalance, residual confounding, or biological heterogeneity. Further prospective studies are needed to clarify the potential sex-specific associations.

OSA is strongly associated with hypertension, and inflammation is considered the key mechanism underlying this relationship. Previous studies have suggested that OSA may contribute to elevated blood pressure through intermittent hypoxia, sympathetic nervous system activation, oxidative stress, endothelial dysfunction, and vascular remodeling.^[31,32]^ Mechanisms including obesity, decreased intrathoracic pressure, activation of pulmonary stretch receptors, chemoreceptor stimulation, hypoxemia, and hypercapnia may further aggravate sympathetic activation and inflammatory responses, ultimately promoting the development of hypertension.

Numerous inflammatory markers, including C-reactive protein (CRP), interleukin-6 (IL-6), interleukin-10 (IL-10), tumor necrosis factor (TNF), and interferon (IFN) have been investigated to better understand the relationship between OSA and systemic inflammation. However, previous studies have reported inconsistent findings regarding changes in these inflammatory markers following OSA treatment.^[31]^ These inconsistencies may partly reflect the complex interactions between obesity, demographic characteristics, OSA severity, and the balance between pro-inflammatory and anti-inflammatory pathways.^[33,34]^ Therefore, the inflammatory mechanisms linking OSA and hypertension remain unclear.

ALI is an innovative composite marker that integrates BMI, serum albumin, and neutrophil-to-lymphocyte ratio (NLR), thereby reflecting both nutritional and inflammatory status. BMI and serum albumin are commonly used indicators of nutritional status,^[35]^ whereas the NLR is recognized as a simple and reliable marker of systemic inflammation. As a widely used indicator of body fat composition, BMI is closely associated with obesity, which is an important risk factor for both OSA and hypertension. Previous studies have demonstrated that reductions in obesity are associated with improvements in OSA severity, highlighting the important role of BMI in OSA progression.^[36]^ In addition, obesity-related sympathetic nervous system overactivation may increase catecholamine secretion, cardiac output, and peripheral vascular resistance, thereby contributing to the development of hypertension. Activation of the renin-angiotensin-aldosterone system and increased endothelin production may also participate in this process.^[37]^

Serum albumin, the most abundant plasma protein, is closely associated with nutritional and inflammatory statuses. Lower albumin levels have been linked to hypertension and adverse cardiovascular outcomes.^[38]^ Furthermore, ischemia-modified albumin levels have been reported to correlate with OSA and subclinical cardiovascular events.^[39]^ The NLR, which represents the ratio of neutrophils to lymphocytes, provides a broader reflection of systemic inflammatory activity than isolated blood cell counts alone. Previous studies have demonstrated significantly elevated neutrophil and lymphocyte levels in patients with OSA, supporting the role of inflammation in OSA pathophysiology.^[40,41]^ Existing meta-analyses have consistently demonstrated elevated NLR levels in hypertensive patients, potentially linked to lymphocyte counts and subtypes. A reduction in lymphocyte numbers not only reflects poor overall health but also triggers a cascade of physiological stress responses.^[42]^ Moreover, the activity of different T lymphocyte subtypes contributes to varying blood pressure profiles.^[43,44]^ By combining these three components, ALI offers an integrated evaluation of nutritional and inflammatory states, enhancing its predictive power. Thus, ALI is a valuable and precise tool for assessing the risk of hypertension in patients with OSA.

This study makes several important contributions to the existing literature. Utilizing a large, nationally representative sample minimizes potential sampling bias and enhances the generalizability of the findings. The NHANES dataset, recognized for its rigorous methodology and high-quality data collection, strengthens the credibility and reliability of the analysis. In addition, this study provides further evidence supporting the association between ALI and hypertension among individuals with OSA, highlighting the potential relevance of inflammatory and nutritional status in this population. These findings may contribute to improving strategies for hypertension assessment and management in OSA patients.

### 4.1. Limitations

However, this study had several limitations. First, the cross-sectional design precludes causal inference, and prospective longitudinal studies are needed to validate these findings further. Second, OSA and several sleep-related variables were identified using self-reported questionnaire data rather than objective assessments, such as polysomnography, which may have introduced recall bias and potential misclassification bias. Although the questionnaire items used to define OSA have been widely applied in epidemiological studies, questionnaire-based symptom classification may not fully capture the presence or severity of OSA and may be less accurate than objective diagnostic methods. Finally, participants with missing data were excluded from the analysis, which may have affected the representativeness of the study population and introduced potential selection bias. Nevertheless, the nationally representative sampling design and large sample size of the NHANES may partially mitigate this issue.

## 5. Conclusion

This study found that ALI was significantly associated with hypertension in individuals with OSA, highlighting the potential relevance of nutritional and inflammatory status in this population.

## Acknowledgments

We thank the NCHS for their efforts in creating the data for the NHANES.

## Author contributions

**Conceptualization:** Mengying Lin, Songtao Li.

**Data curation:** Mengying Lin, Yuxin Yang, Zongshan Li, Tian Lv.

**Formal analysis:** Mengying Lin, Zongshan Li, Shaoting Yu.

**Visualization:** Mengying Lin, Zongshan Li, Shaoting Yu, Xiaoyan Yang.

**Writing – original draft:** Mengying Lin, Yuxin Yang, Zongshan Li, Tian Lv, Shaoting Yu, Xiaoyan Yang.

**Investigation:** Yuxin Yang, Zongshan Li, Shaoting Yu, Xiaoyan Yang.

**Methodology:** Yuxin Yang, Songtao Li, Jia Huang.

**Validation:** Yuxin Yang, Songtao Li.

**Writing – review & editing:** Yuxin Yang, Tian Lv, Yourang Pan, Songtao Li, Jia Huang.

**Software:** Zongshan Li, Tian Lv, Yourang Pan.

**Funding acquisition:** Tian Lv, Yourang Pan.

**Resources:** Yourang Pan, Jia Huang.

**Project administration:** Jia Huang.

**Supervision:** Jia Huang.


