# Supplementary material for: Association of advanced lung cancer inflammation index with hypertension in obstructive sleep apnea
Source: Medicine (Baltimore). 2026 Sep 11;105(37):e50538. doi: 10.1097/MD.0000000000050538 (PMC13574479; doi:10.1097/MD.0000000000050538)
Supplement: Supplementary file 1 [file medi-105-e50538-s001.docx]

Table 2 Sensitivity Analyses

| Exposure | Model 1 | | Model 2 | | Model 3 | |
| --- | --- | --- | --- | --- | --- | --- |
|  | 95%CI | P | 95%CI | P | 95%CI | P |
| ALI/100 | 1.40(1.20,1.64) | <0.001 | 1.31(1.12,1.54) | 0.002 | 1.32(1.10,1.60) | 0.01 |
| ALI for quartiles | | | | | | |
| Q1 | ref |  | ref |  | ref |  |
| Q2 | 1.19(0.99,1.43) | 0.06 | 1.19(0.99,1.43) | 0.06 | 1.29(1.05,1.58) | 0.02 |
| Q3 | 1.26(1.02,1.55) | 0.03 | 1.23(1.01,1.51) | 0.04 | 1.25(0.99,1.58) | 0.06 |
| Q4 | 1.73(1.41,2.12) | <0.0001 | 1.58(1.27,1.97) | <0.001 | 1.63(1.26,2.10) | 0.002 |
| p for trend |  | <0.0001 |  | <0.001 |  | 0.001 |

Model 1: adjusted for age and sex.

Model 2: adjusted for included crude model, age, sex, education and race.

Model 3: adjusted for all model 2 in addition to smoking status, alcohol status, diabetes, hyperlipidemia, WBC and eGFR.
